# MicroRNA Signatures of Fulvestrant-Treated Luminal Breast Cancer Cells: Identification of Therapeutic Targets Regulated by *miR-374b-5p*

**DOI:** 10.3390/ijms27156787

**Published:** 2026-07-29

**Authors:** Ayako Nagata, Yuya Tomioka, Ryutaro Yasudome, Hiroko Toda, Takuya Tokunaga, Yuki Nagata, Mayuko Kato, Yoshiaki Shinden, Akihiro Nakajo, Naohiko Seki

**Affiliations:** 1 Department of Breast and Thyroid Surgery, Graduate School of Medical and Dental Sciences, Kagoshima University, Kagoshima 890-8544, Japan; k8235150@kadai.jp (A.N.); k7682205@kadai.jp (R.Y.); k0848362@kadai.jp (H.T.); k4483271@kadai.jp (Y.S.); anakajo@m.kufm.kagoshima-u.ac.jp (A.N.); 2 Department of Pulmonary Medicine, Graduate School of Medical and Dental Sciences, Kagoshima University, Kagoshima 890-8544, Japan; k4829264@kadai.jp; 3 Department of General Thoracic Surgery, Graduate School of Medical and Dental Sciences, Kagoshima University, Kagoshima 890-8544, Japan; k1933430@kadai.jp; 4 Department of Digestive Surgery, Graduate School of Medical and Dental Sciences, Kagoshima University, Kagoshima 890-8544, Japan; k7989794@kadai.jp; 5 Department of Functional Genomics, Chiba University Graduate School of Medicine, Chuo-ku, Chiba 260-8670, Japan; mayukokato@chiba-u.jp

**Keywords:** breast cancer, estrogen receptor, fulvestrant, microRNA, *miR-374b-5p*, *FOXM1*

## Abstract

Estrogen receptor (ER)-positive breast cancer (BrCa) accounts for two-thirds of all BrCa cases worldwide. Therefore, ER-targeted endocrine therapy is the standard treatment for this disease. There has been a recent trend towards developing combination therapies using molecularly targeted drugs to improve outcomes. This study aimed to identify therapeutic targets demonstrating efficacy when combined with fulvestrant (a selective ER downregulator/degrader). We generated microRNA (miRNA) signatures from fulvestrant-treated MCF-7 cells by RNA sequencing. From the signature, we evaluated *miR-374b-5p* because its expression was elevated by fulvestrant treatment in MCF-7 cells. Also, in expression analysis by subtype of BrCa patients, *miR-374b-5p* expression was suppressed only in luminal BrCa. Ectopic expression assays revealed that *miR-374b-5p* attenuated the malignant phenotypes of MCF-7 cells. We searched for genes regulated by *miR-374b-5p* and discovered that 11 (*NEK2*, *NUF2*, *HMMR*, *DEPDC1B*, *FOXM1*, *ELOVL6*, *KIF20A*, *NCAPH*, *CENPK*, *FAM83D*, and *KIAA0101*) are closely involved in BrCa molecular pathogenesis. Among these target genes, we focused on *forkhead box M1* (*FOXM1*), a transcription factor regulating cell cycle progression and division. Notably, combination therapy with fulvestrant and a *FOXM1* inhibitor significantly suppressed MCF-7 cell proliferation. From the miRNA signature established in this study, we identified antitumor *miR-374b-5p* and its target genes and used these findings to explore candidate drugs with potential efficacy when combined with fulvestrant.

## 1. Introduction

Breast cancer (BrCa) is one of the most significant malignancies affecting women worldwide, with approximately 2.3 million new cases and 670,000 deaths estimated globally in 2022 [1]. Breast cancer is currently classified into several molecular subtypes, including luminal A, luminal B, HER2-enriched/positive, and basal-like/triple-negative subtypes, and treatment strategies and clinical outcomes differ according to subtype [2,3]. Both the luminal A and luminal B subtypes strongly express the estrogen receptor (ER). ER-positive BrCa accounts for approximately 70% of all BrCa cases diagnosed worldwide [4,5]. Additionally, luminal A BrCa is generally characterized by ER positivity, HER2 negativity, high progesterone receptor expression, and a low proliferation index. Approximately 30–40% of invasive BrCa cases are classified as luminal A [6].

Endocrine therapy is a primary treatment for ER-positive BrCa. Currently, selective ER modulators, selective ER downregulators/degraders, and aromatase inhibitors are used as therapeutic agents for such cases [5,7]. For luminal A-type patients, endocrine therapy remains the foundation of systemic therapy. However, resistance inevitably arises due to genetic alterations and activating oncogenic pathways, limiting its long-term effectiveness, and in many cases, the cancer progresses to an advanced stage [8,9].

Fulvestrant, a selective ER downregulator/degrader, is a novel ER antagonist that competitively binds to the ER with much higher affinity than tamoxifen. The reduction in the intracellular level of ER protein inhibits the transcription of estrogen-inducible genes [10,11]. Currently, not only fulvestrant monotherapy but also combination therapy with other molecularly targeted drugs is being used clinically [12,13]. We hypothesized that suppressed expression of tumor-suppressive genes by ER signaling would be restored by fulvestrant treatment in BrCa cells.

In the post-genome sequencing era, it has become clear that non-protein-coding RNAs are transcribed in large quantities within cells and play important roles in various physiological processes [14]. Unraveling the extremely complex RNA networks (protein-coding and noncoding RNAs) within human cells is a major goal. Among the noncoding RNAs, microRNAs (miRNAs) are short, single-stranded, functional RNAs whose importance in cancer cells has been reported in numerous studies [15]. A key biological characteristic of miRNAs is that a single miRNA controls the expression of numerous protein-coding genes within a cell [16]. Therefore, aberrant expression of miRNAs can have a serious impact on the expression of protein-coding genes, disrupting the intracellular RNA network in cells [16]. With advances in RNA sequencing technology, miRNA expression profiles have been generated for every cancer type, and characteristic abnormalities in miRNA expression have been reported for each cancer type [17].

We have previously reported miRNA expression signatures using RNA sequencing of BrCa clinical specimens [18]. Furthermore, from these signatures, we identified tumor-suppressive miRNAs and their regulated oncogenic targets in BrCa cells [19,20]. For example, *miR-30c-3p* exerts tumor-suppressive functions in BrCa cells by controlling several genes closely involved in BrCa malignant phenotypes, such as *TRIP13*, *CCNB1*, *RAD51*, and *CENPN* [19]. Notably, a specific inhibitor of *TRIP13*, one of these *miR-30c-3p* target genes, suppressed the malignant transformation of BrCa cells [19].

In this study, we profiled changes in miRNA expression in fulvestrant-treated MCF-7 cells using RNA sequencing. This signature contained many miRNAs (e.g., *miR-139-3p*, *miR-30a-3p*, *miR-30c-2-3p*, and *miR-26a-5p*) that we had previously reported to be tumor suppressive in various cancers [19,20,21,22]. We focused on *miR-374b-5p*, which has not been reported in many BrCa studies. In expression analysis by subtype of BrCa patients, *miR-374b-5p* expression was suppressed only in luminal BrCa. To date, there have been few studies analyzing *miR-374b-5p* in luminal breast cancer cells. Therefore, exploring the function of *miR-374b-5p* and its regulated genes will be helpful in understanding the molecular mechanisms of luminal BrCa. Accordingly, we investigated its antitumor functions and target genes.

Ectopic expression assays showed that *miR-374b-5p* had antitumor functions in MCF-7 cells. A total of 11 genes (*NEK2*, *NUF2*, *HMMR*, *DEPDC1B*, *FOXM1*, *ELOVL6*, *KIF20A*, *NCAPH*, *CENPK*, *FAM83D*, and *KIAA0101*) were identified as potential therapeutic targets of antitumor *miR-374b-5p* in BrCa cells. These genes are closely involved in the molecular pathogenesis of luminal A BrCa. Interestingly, a *FOXM1* (*forkhead box M1*) inhibitor (FDI-6), when used in combination with fulvestrant, significantly suppressed MCF-7 cell proliferation in vitro. Our miRNA-based analysis is an effective strategy for discovering drugs that show potential synergistic effects with endocrine therapy.

## 2. Results

### 2.1. Identification of miRNAs with Upregulated Expression in Fulvestrant-Treated MCF-7 Cells According to RNA Sequencing

Advances in RNA sequencing technology have enabled rapid and accurate generation of short, single-stranded miRNA expression signatures. In this study, MCF-7 cells were treated with fulvestrant to identify the miRNAs upregulated in expression.

At 72 h after fulvestrant treatment, the expression of 35 miRNAs was upregulated compared with untreated cells (Table 1).

**Figure 1:**
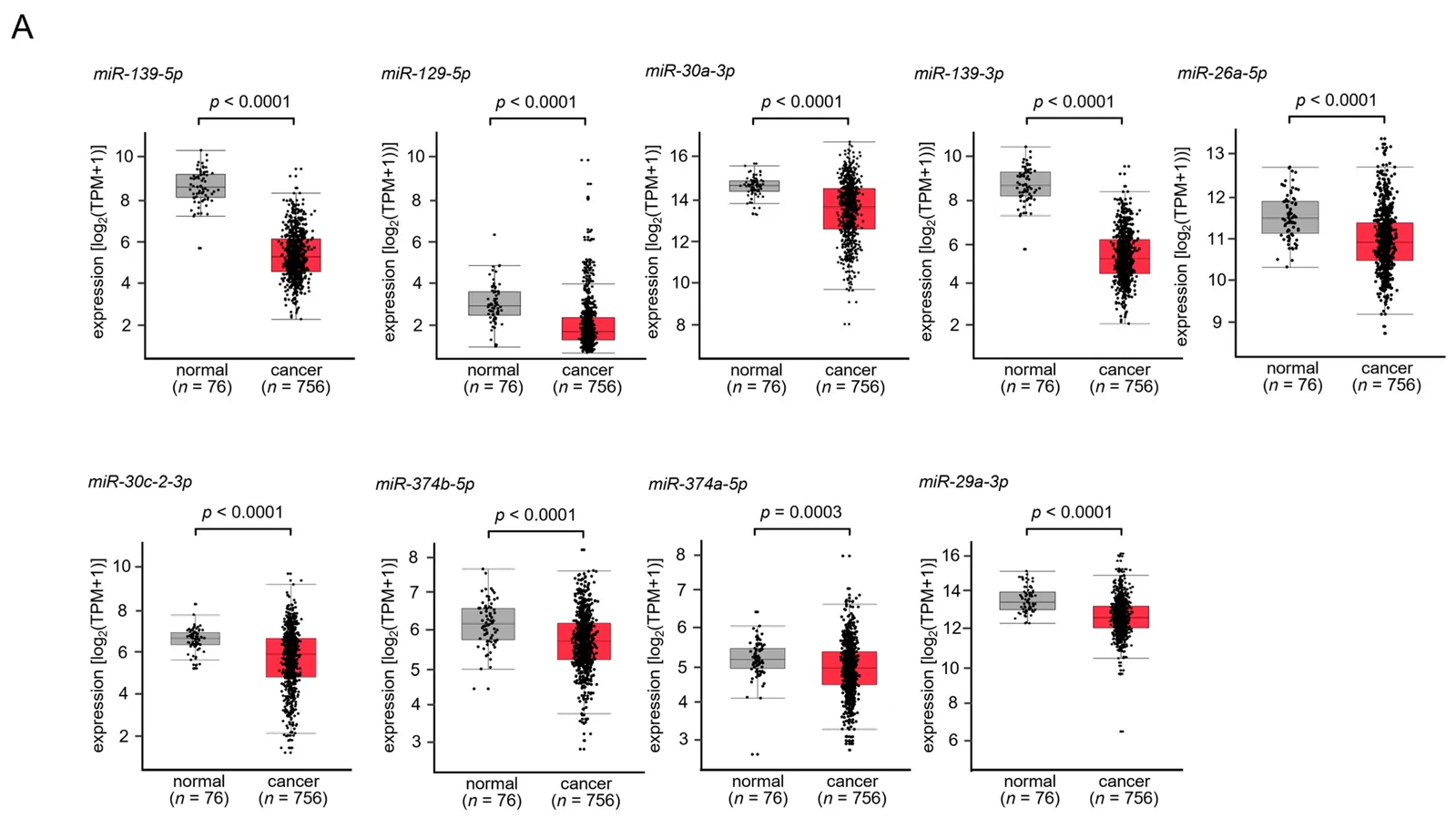

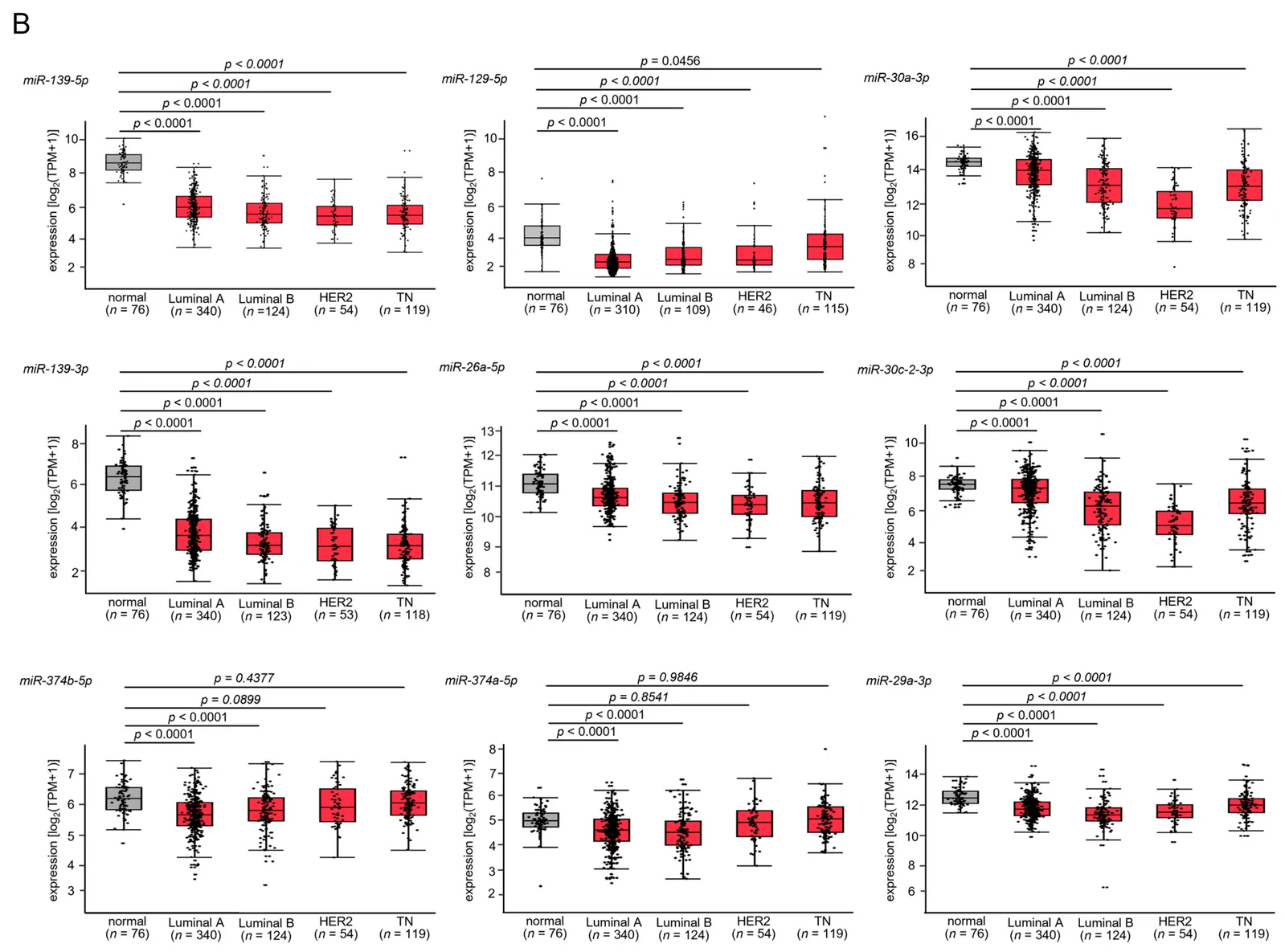
Expression levels of nine miRNAs (*miR-139-5p*, *miR-129-5p*, *miR-30a-3p*, *miR-139-3p*, *miR-26a-5p*, *miR-30c-2-3p*, *miR-374b-5p*, *miR-374a-5p*, and *miR-29a-3p*) in breast cancer tissues: (**A**) All nine miRNAs were significantly downregulated in breast cancer tissues (*n* = 756) compared with normal tissues (*n* = 76). (**B**) The expression levels of the nine miRNAs were analyzed in each subtype of breast cancer (luminal A, luminal B, HER2-positive (HER2), and triple-negative (TN)).

Furthermore, we analyzed a BRCA dataset from TCGA to determine which of these 35 miRNAs are tumor suppressive. TCGA analysis revealed that nine of the miRNAs (*miR-139-5p*, *miR-129-5p*, *miR-30a-3p*, *miR-139-3p*, *miR-26a-5p*, *miR-30c-2-3p*, *miR-374b-5p*, *miR-374a-5p*, and *miR-29a-3p*) are significantly downregulated in BrCa tissue compared with normal tissue (Figure 1A).

Next, the expression of the nine miRNAs was analyzed in BrCa tissues according to subtype: luminal A, luminal B, HER2-positive, and triple-negative. Interestingly, *miR-374b-5p* and *miR-374a-5p* expression was suppressed only in the luminal A and luminal B subtypes compared with normal breast tissues (Figure 1B). Other miRNAs were suppressed in all subtypes compared with normal breast tissues (Figure 1B). In this study, we focused our analysis on *miR-374b-5p* in MCF-7 cells.

### 2.2. Functional Assays of miR-374b-5p Ectopically Expressed in MCF-7 Cells

**Figure 2:**
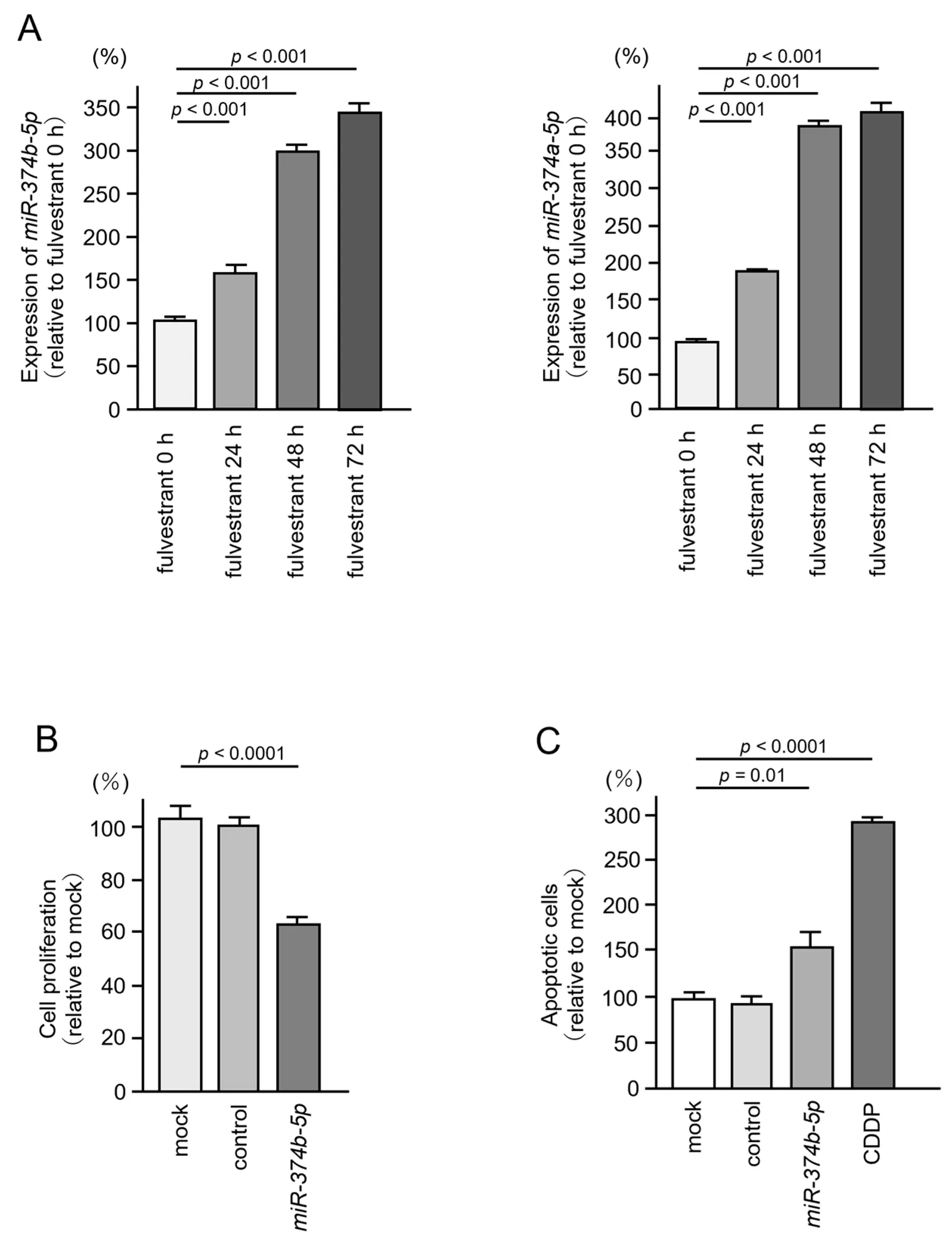
Tumor-suppressive roles of *miR-374b-5p* in MCF-7 cells: (**A**) *miR-374b-5p* expression was increased in a time-dependent manner in MCF-7 cells. The final concentration of *miR-374b-5p* was 10 nM. (**B**) Cell proliferation was assessed by XTT assay at 72 h after transfection with *miR-374b-5p* in MCF-7 cells. (**C**) Apoptotic cells were evaluated by flow cytometry 72 h after transfection with *miR-374b-5p* in MCF-7 cells. Cisplatin (CDDP) was used as a positive control for apoptosis induction.

We further analyzed *miR-374b-5p* and *miR-374a-5p*, which we have not analyzed in any cancer type previously. Expression of *miR-374b-5p* and *miR-374a-5p* was increased by fulvestrant treatment in a concentration- and time-dependent manner (Figure 2A). Since the mature sequences of *miR-374b-5p* and *miR-374a-5p* differ by only two nucleotides and share identical seed sequences, subsequent analyses were performed using *miR-374b-5p* only.

To investigate the functional roles of *miR-374b-5p*, we overexpressed it in MCF-7 cells and then performed cell proliferation and apoptosis assays using these cells. Overexpression of *miR-374b-5p* significantly suppressed cell proliferation, as determined by the XTT assay (Figure 2B). Furthermore, the apoptosis assay demonstrated a significant increase in the percentage of apoptotic cells in *miR-374b-5p*-transfected cells compared with negative control miRNA-transfected cells (Figure 2C). These results suggest that *miR-374b-5p* exerts tumor-suppressive functions by inhibiting growth and stimulating apoptosis in MCF-7 cells.

### 2.3. Identification of miR-374b-5p Target Genes in MCF-7 Cells

**Figure 3:**
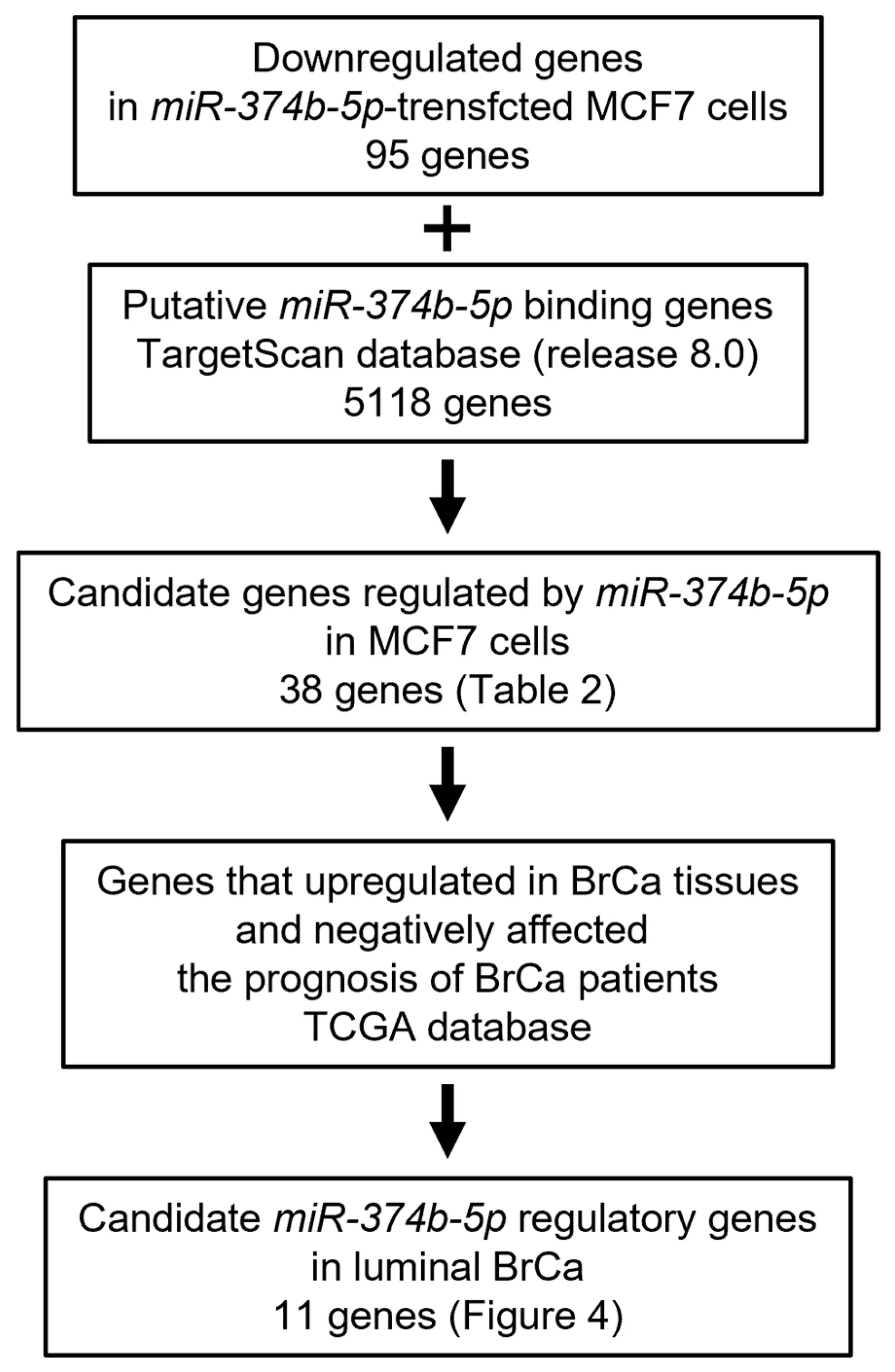
Identification of *miR-374b-5p* oncogenic targets in MCF-7 cells. Flowchart for the identification of *miR-374b-5p* oncogenic targets in MCF-7 cells. Of the 95 genes with significantly suppressed expression in *miR-374b-5p*-transfected MCF-7 cells, 38 had *miR-374b-5p*-binding sequences. TCGA analysis revealed that 11 of these genes are upregulated in breast cancer tissue, and the expression of these mRNAs was negatively associated with patient prognosis.

Next, we investigated the genes regulated by tumor-suppressive *miR-374b-5p* in MCF-7 cells. Figure 3 shows the flowchart of the search process for *miR-374b-5p* target genes performed in this study.

First, we searched for genes with suppressed expression in *miR-374b-5p*-transfected MCF-7 cells. RNA sequencing of these cells revealed that 95 genes were significantly suppressed in expression. Next, a search of the TargetScan database (release 8.0) revealed that 5118 genes contain *miR-374b-5p*-binding sequences in their 3′ UTR. Merging the results from these two analyses identified 38 genes as potential target genes for *miR-374b-5p* (Table 2).

### 2.4. Identification of Therapeutic Targets in BrCa

**Figure 4:**
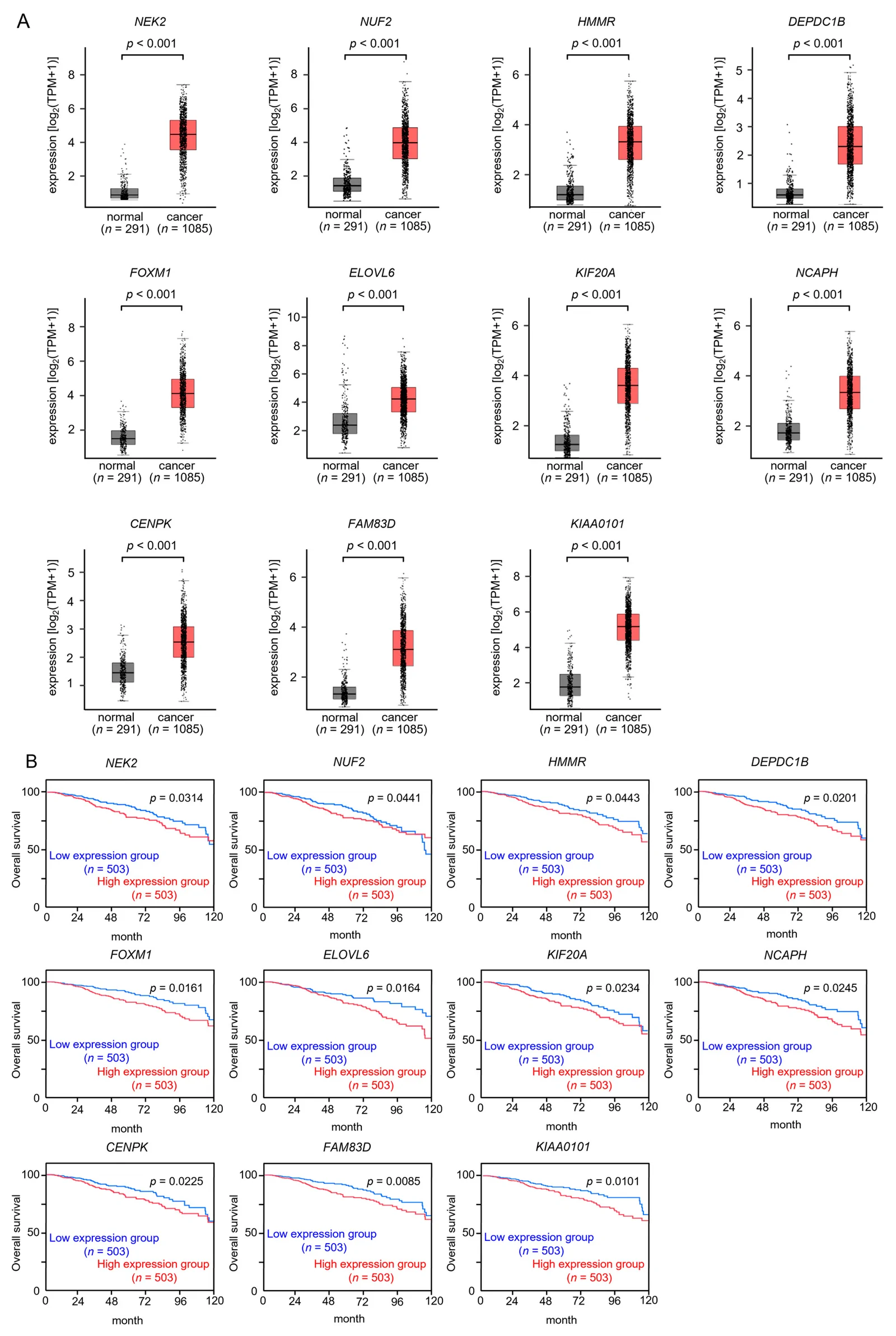

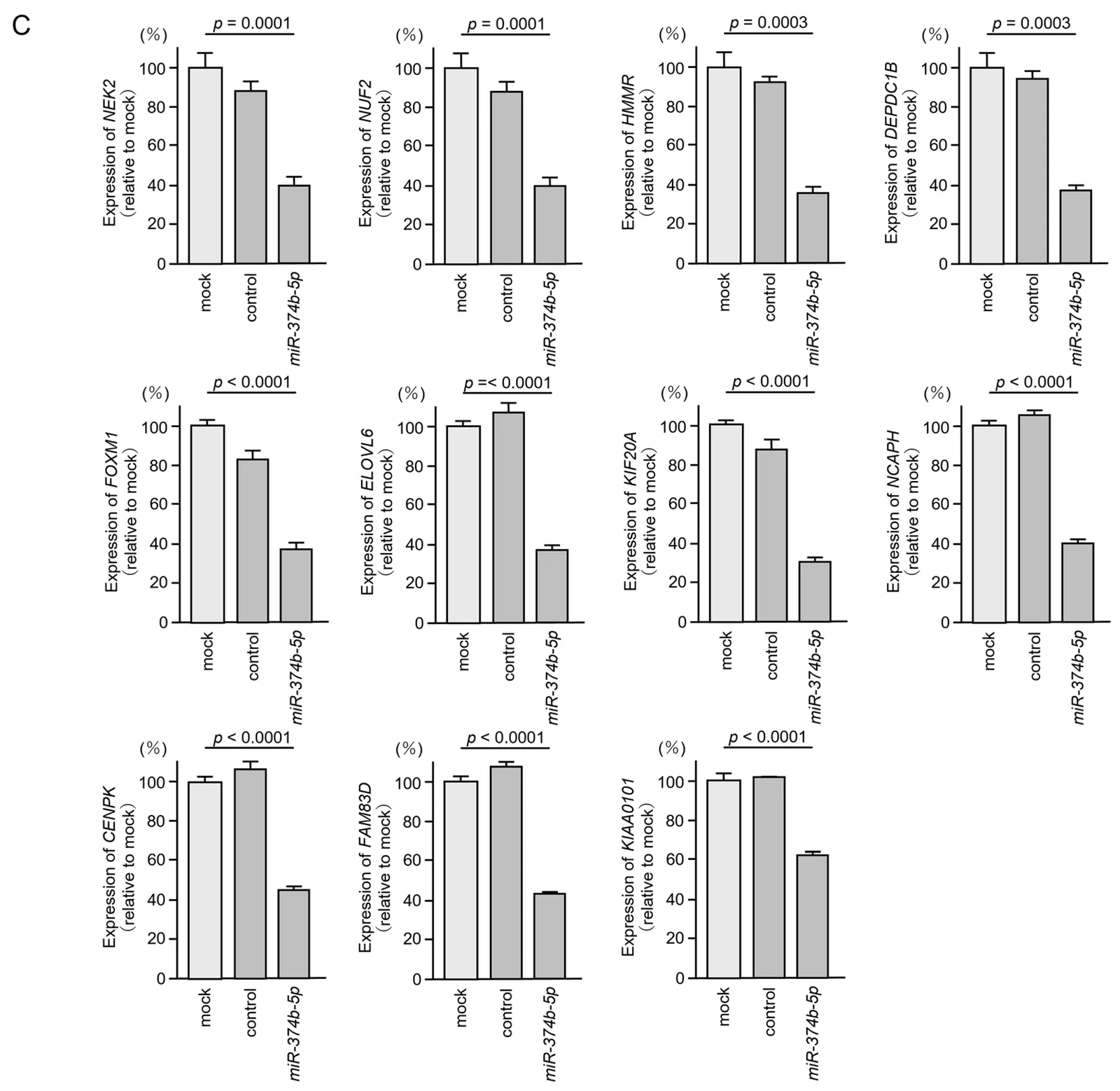
Expression and clinical features of 11 genes (*NEK2*, *NUF2*, *HMMR*, *DEPDC1B*, *FOXM1*, *ELOVL6*, *KIF20A*, *NCAPH*, *CENPK*, *FAM83D*, and *KIAA0101*) according to breast cancer data from TCGA: (**A**) All genes were upregulated in breast cancer tissues (*n* = 1085) compared with normal tissues (*n* = 291). (**B**) Kaplan–Meier curves are shown for the 10-year overall survival rate based on the expression of 11 genes according to breast cancer data from TCGA. Patients (*n* = 1006) were stratified into high- and low-expression groups based on the median expression level of each gene. The red and blue lines represent the high- and low-expression groups, respectively. (**C**) The expression levels of the 11 genes (*NEK2*, *NUF2*, *HMMR*, *DEPDC1B*, *FOXM1*, *ELOVL6*, *KIF20A*, *NCAPH*, *CENPK*, *FAM83D*, and *KIAA0101*) were significantly reduced in *miR-374b-5p*-transfected MCF-7 cells compared with the parental cells.

We searched for therapeutic targets for BrCa among the 38 *miR-374b-5p* target genes using a BRCA dataset from TCGA. We identified genes that met the following two conditions: upregulated expression in BrCa tissues and an adverse effect of its expression on patient prognosis. After applying these criteria, 11 genes remained: *NEK2*, *NUF2*, *HMMR*, *DEPDC1B*, *FOXM1*, *ELOVL6*, *KIF20A*, *NCAPH*, *CENPK*, *FAM83D*, and *KIAA0101* (Figure 4A,B).

The expression of these target genes was analyzed separately in each BrCa subtype. All genes were upregulated in each BrCa subtype compared with normal breast tissues (Supplemental Figure S1), with the only exception being that *ELOVL6* was not upregulated in the HER2-positive subtype (Supplemental Figure S1). We also confirmed that the expression levels of all 11 genes were significantly suppressed in *miR-374b-5p*-transfected MCF-7 cells (Figure 4C).

### 2.5. Regulation of FOXM1 by miR-374b-5p in MCF-7 Cells

Among the 11 *miR-374b-5p* target genes, we focused on *FOXM1* because it is a transcription factor that regulates cell division and the transition from G2 to M phase in the cell cycle.

**Figure 5:**
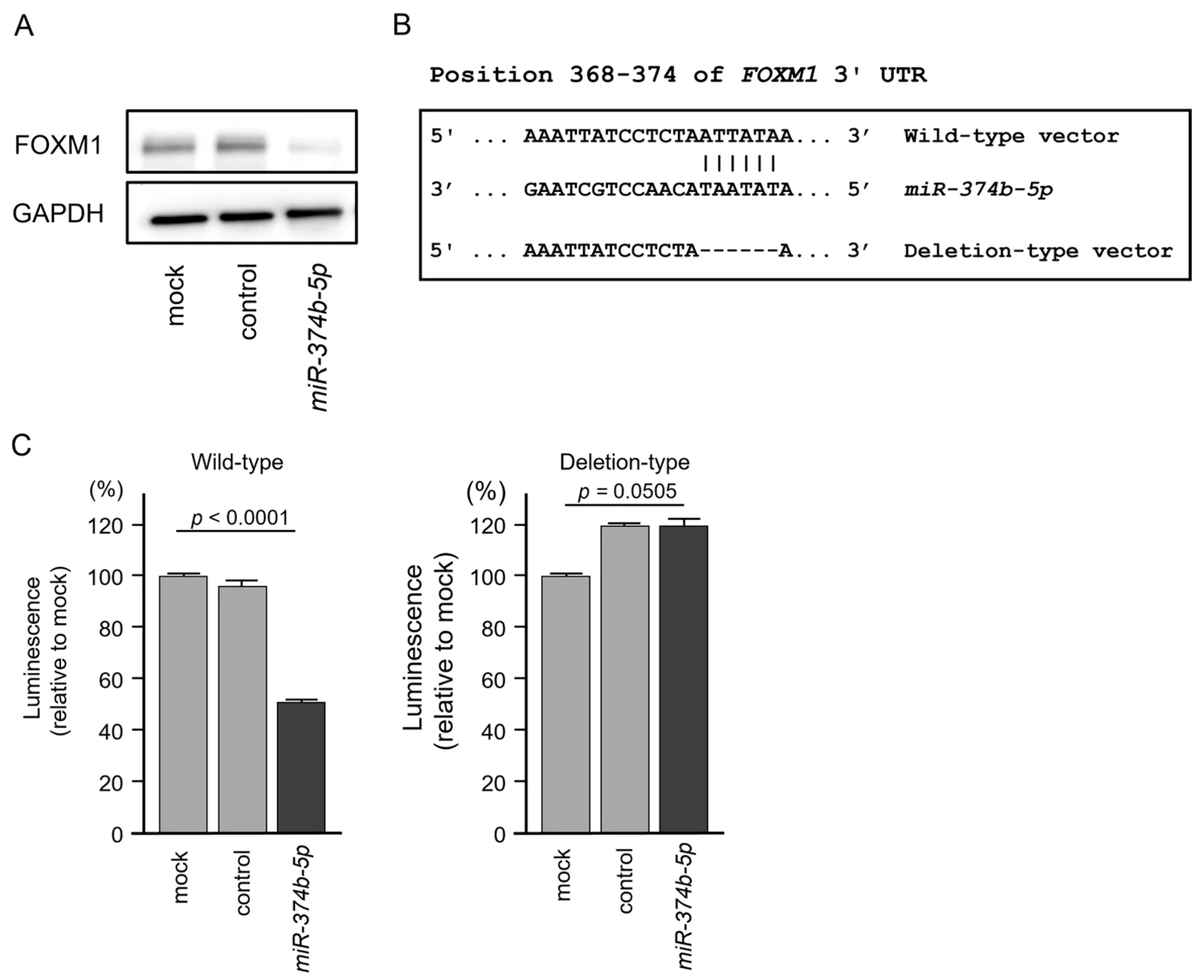
Direct regulation of *FOXM1* by *miR-374b-5p* in MCF-7 cells: (**A**) Expression of the FOXM1 protein level was markedly reduced in *miR-374b-5p*-transfected MCF-7 cells. Protein samples were collected at 72 h after *miR-374b-5p* transfection and quantified by Western blotting. GAPDH was used as the internal control. (**B**) The annotated *miR-374b-5p*-binding site in the 3′ UTR of *FOXM1* was identified by analysis of the TargetScanHuman database (release 8.0). (**C**) Dual-luciferase reporter assays revealed reduced luminescence activity in MCF-7 cells co-transfected with *miR-374b-5p* and a vector containing the wild-type *miR-374b-5p*-binding site from the *FOXM1* 3′ UTR. In contrast, no such reduction in luminescence activity was observed after co-transfection with a construct containing a deletion in the *miR-374b-5p*-binding site.

First, we found that *FOXM1* mRNA and protein levels were significantly suppressed by ectopic expression of *miR-374b-5p* in MCF-7 cells (Figure 4C and Figure 5A). The full-size Western blot is shown in Supplemental Figure S2.

Next, we performed luciferase reporter assays to determine whether *miR-374b-5p* binds directly to its target sequence within *FOXM1*. The annotated binding sequence of *miR-374b-5p* in the 3′ UTR of *FOXM1* is shown in Figure 5B. Luminescence activity was suppressed in cells transfected with a vector carrying the wild-type *miR-374b-5p*-binding sequence (Figure 5C). On the other hand, luminescence activity was not suppressed in cells transfected with a vector carrying the 3′ UTR of *FOXM1* in which the *miR-374b-5p*-binding sequence was deleted (Figure 5C). The full sequences that were cloned into the vectors are shown in Supplemental Figure S3.

### 2.6. Inhibition of Cell Proliferation by Combination Therapy with Fulvestrant and the FOXM1 Inhibitor FDI-6

**Figure 6:**
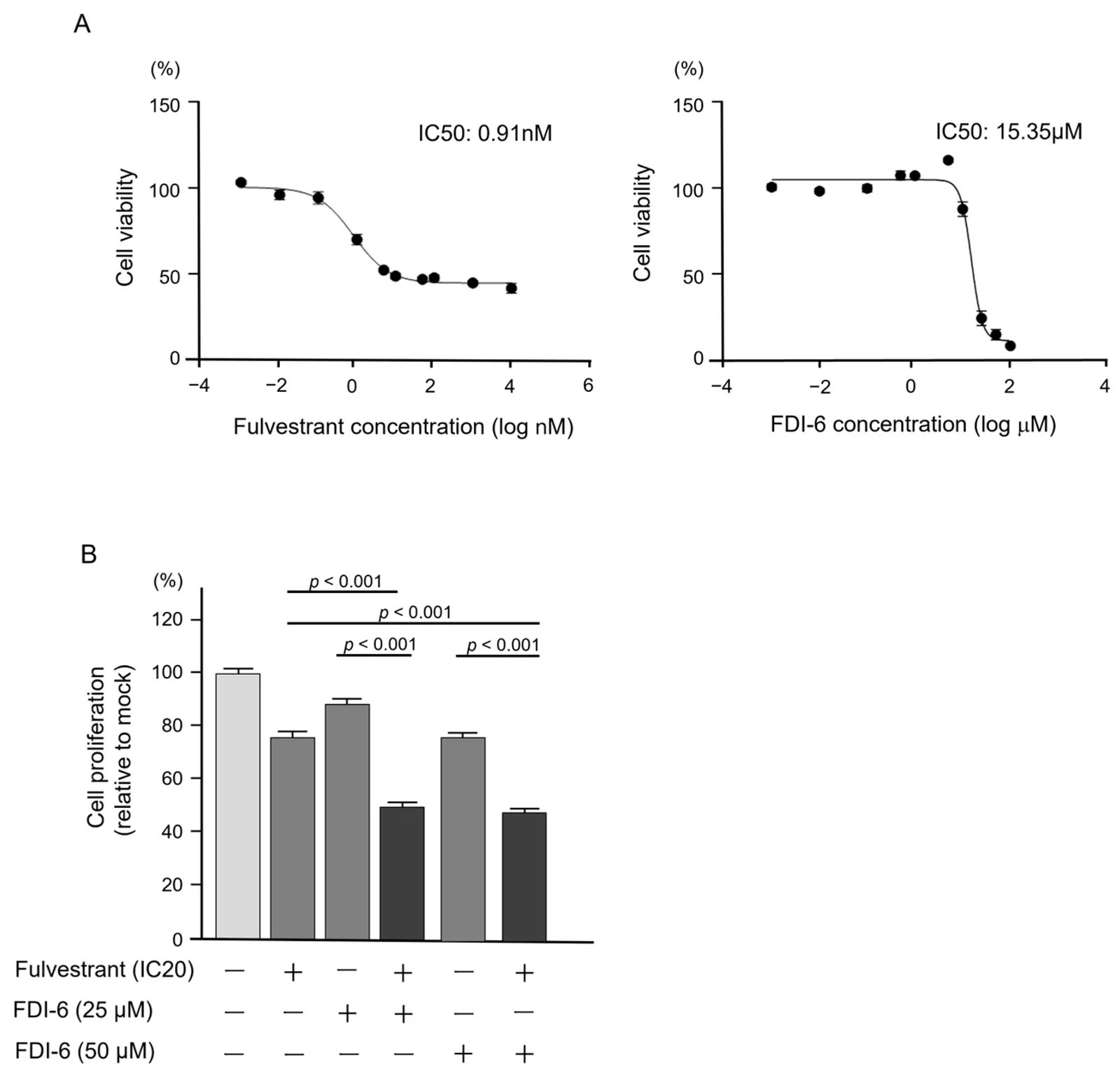
Effect of co-treatment with a FOXM1 inhibitor (FDI-6) and fulvestrant in MCF-7 cells: (**A**) The proliferation of MCF-7 cells was slightly inhibited by fulvestrant or the FOXM1 inhibitor FDI-6. (**B**) The proliferation of MCF-7 cells was significantly inhibited by the combination of fulvestrant plus FDI-6 compared with either monotherapy.

The antitumor effects of fulvestrant and FDI-6, a *FOXM1* inhibitor, were evaluated in MCF-7 cells. The IC50 values of fulvestrant and FDI-6 in MCF-7 cells were 0.91 nM and 15.35 µM, respectively (Figure 6A). Cell proliferation was significantly suppressed by treatment with fulvestrant (IC20) combined with FDI-6 (25 µM or 50 µM) compared with fulvestrant monotherapy (Figure 6B).

## 3. Discussion

For patients with ER-positive/HER2-negative BrCa who are not candidates for surgery or who have metastasis, the first-line treatment is combination therapy with endocrine therapy and CDK4/6 inhibitors (abemaciclib and palbociclib) [23]. The medications used for endocrine therapy will depend on the patient’s past medication history [24].

Fulvestrant, a selective ER downregulator/degrader, is used in combination with a CDK4/6 inhibitor in patients with hormone receptor-positive/HER2-negative metastatic BrCa who relapse during adjuvant aromatase inhibitor therapy or within 12 months after completion of adjuvant endocrine therapy [25].

Fulvestrant is a selective ER degrader/downregulator that promotes ER degradation and thereby blocks ER-mediated signaling. Fulvestrant has been used for patients with hormone receptor-positive advanced BrCa who experience disease progression following endocrine therapy [26]. Acquisition of *ESR1* (the gene encoding ERα) mutations is one reported mechanism of endocrine therapy resistance, and treatments for patients with *ESR1* mutations are being developed [27]. *ESR1* mutations are rarely detected in primary or endocrine therapy-naïve BrCa, whereas they are frequently observed in metastatic BrCa after exposure to aromatase inhibitors [28]. For BrCa patients with *ESR1* mutations, drugs with ER-degradation effects may improve patient prognosis.

Activation of oncogenic PI3K/AKT/mTOR signaling is frequently detected in a wide range of human cancers [29]. More than half of ER-positive/HER2-negative BrCa patients have alterations in genes involved in this pathway, and these alterations are the cause of resistance to endocrine therapy [30]. Capivasertib is a potent competitive inhibitor of the *AKT* kinase that inhibits all three *AKT* isoforms. It blocks activation of *AKT* signaling by suppressing the phosphorylation of downstream effector molecules [31].

Combination therapy with capivasertib and fulvestrant was approved in 2023 for the treatment of ER-positive/HER2-negative BrCa with PI3K/AKT/PTEN gene alterations [13]. The discovery of therapeutic targets for BrCa that can be combined with fulvestrant to improve patient prognosis is a crucial challenge, and exploration using various approaches continues.

In this study, we identified miRNAs whose expression was upregulated in MCF-7 cells after fulvestrant treatment (presumably by blocking ER-mediated oncogenic signaling). Of the miRNAs affected by fulvestrant, *miR-30a-3p* and *miR-30c-2-3p* were previously reported by our group to be tumor-suppressive miRNAs in BrCa cells [19,20]. We also reported that *ANLN* (actin-binding protein) and *TRIP13* (thyroid hormone receptor interactor 13) are therapeutic targets for BrCa and are directly regulated by *miR-30a-3p* and *miR-30c-2-3p*, respectively [19,20]. *miR-139-5p* showed the greatest increase in expression after fulvestrant treatment in this study, and it has been reported to have antitumor functions in many cancers, including BrCa [32]. The expression of *miR-139-3p*, the passenger strand derived from the *miR-139* precursor, was also elevated by fulvestrant treatment. We previously revealed the oncogenes regulated by *miR-139-3p* in multiple cancer types, such as lung adenocarcinoma, colorectal cancer, head and neck squamous cell carcinoma, and renal cell carcinoma [21,33,34,35]. Searching for oncogenes regulated by both *miR-139-5p* and *miR-139-3p* could lead to the discovery of new therapeutic target molecules for BrCa.

From our miRNA signatures, we focused on *miR-374b-5p* because its functional significance and target genes in BrCa are largely unknown. We showed that *miR-374b-5p* acted as an antitumor miRNA and regulated the expression of 11 genes *(NEK2*, *NUF2*, *HMMR*, *DEPDC1B*, *FOXM1*, *ELOVL6*, *KIF20A*, *NCAPH*, *CENPK*, *FAM83D*, and *KIAA0101*).

Of these genes, *NEK2* (*never in mitosis gene a-related kinase 2*), a serine/threonine protein kinase, plays a central role in mitosis and plays a crucial role in cell cycle regulation and DNA damage repair [36]. Previous reports indicated that *NEK2* inhibition slows cell cycle progression and significantly increases DNA damage and apoptosis [36]. Overexpression of *NEK2* has been reported to induce chromosomal instability and participate in the malignant progression of various cancers, including BrCa [37,38]. Therefore, inhibiting *NEK2* is a goal of cancer treatment.

Aberrant expression of *KIF20A* (*kinesin family member 20A*) has been reported in multiple cancers, including BrCa, making it a promising biomarker for predicting cancer progression and drug therapy [39,40]. Numerous studies have reported that overexpression of kinesin, a motor protein involved in intracellular transport, is intricately involved in cancer progression and drug resistance [41,42]. The JAK/STAT3 pathway is an intracellular signaling mechanism crucial for various cell maintenance processes. Aberrant activation of this pathway is frequently observed in many cancers and is closely involved in cancer progression, metastasis, and drug resistance [43,44]. Increased *KIF20A* expression activates the JAK2/STAT3 pathway by promoting phosphorylation of *JAK2* and *STAT3* [45]. In diffuse large B-cell lymphoma cells, *KIF20A* was reported to be one of the genes controlled by fulvestrant therapy [46]. *KIF20A*, which is controlled by ER-mediated signaling, may be closely involved in resistance mechanisms to endocrine therapy.

Interestingly, the promoter region of *KIF20A* contains a binding element (forkhead response element) for the transcription factor *FOXM1*, and *FOXM1* enhances the transcriptional activity of *KIF20A* [47].

*FOXM1*, functioning as a transcription factor, directly or indirectly regulates the expression of numerous target genes. Disruptions to target genes caused by aberrant expression of *FOXM1* are involved in the malignant transformation of almost all cancers [48]. It has been reported that suppressing *FOXM1* expression enhances drug sensitivity in various cancers. Therefore, the combination therapy of *FOXM1*-targeted inhibitors and anticancer drugs may represent a new treatment strategy for cancers that have acquired drug resistance [48,49].

Aberrant expression of *FOXM1* in BrCa is closely involved in cancer malignancy (e.g., proliferation, invasion, metastasis, and chemotherapy drug resistance) [50,51], and its overexpression is associated with a poor prognosis and clinicopathological characteristics in BrCa patients [50,52]. In BrCa cells, *FOXM1* is a transcriptional target of ERα. Suppressing *FOXM1* expression in MCF-7 cells almost completely suppressed ERα expression [53,54]. Overexpression of *FOXM1* activates estrogen signaling in ER-positive BrCa cells, leading to resistance to anti-estrogen therapy [53,55]. Therefore, *FOXM1* is a pivotal therapeutic target for BrCa. We also investigated the efficacy of combination therapy with fulvestrant and FDI-6 (a specific *FOXM1* inhibitor). The combination therapy significantly suppressed MCF-7 cell proliferation compared with either fulvestrant or FDI-6 monotherapy. *FOXM1* and other *miR-374b-5p* target genes may be potential therapeutic targets for ER-positive BrCa when combined with ER inhibitors.

This study demonstrated that miRNA-based approaches can provide valuable information for elucidating the molecular mechanisms of luminal BrCa cells. Our research group is continuing similar analyses of drugs used in clinical practice for luminal BrCa (e.g., tamoxifen, anastrozole, letrozole). By integrating these analytical data, we expect to identify novel therapeutic targets for this disease.

## 4. Materials and Methods

### 4.1. BrCa Cell Line

The human BrCa cell line MCF-7 was obtained from the JCRB Cell Bank (JCRB0134, Osaka, Japan). MCF-7 cells were cultured in Minimum Essential Medium (MEM) with Earle’s salts and L-glutamine (Nacalai Tesque, Kyoto, Japan; Cat. No. 21442-25), supplemented with 10% fetal bovine serum (Gibco, Thermo Fisher Scientific, Waltham, MA, USA; Cat. No. A5256701) and 1% MEM Non-Essential Amino Acids Solution (100×) (Gibco, Thermo Fisher Scientific, Waltham, MA, USA; Cat. No. 11140050).

### 4.2. Construction of an miRNA Expression Signature Using RNA Sequencing

MCF-7 cells were treated with fulvestrant (S1191; Selleck Chemicals, Houston, TX, USA), and cells were collected at 0, 24, 48, or 72 h after treatment. RNA was extracted from the cells as described previously [19,20]. The RNA samples were sequenced using the Illumina NextSeq 500 system (Illumina, San Diego, CA, USA). The resulting sequencing reads were mapped to the human reference genome, and the miRNA reads were annotated using the miRBase database (https://www.mirbase.org, accessed on 15 September 2025). The process of creating miRNA expression signatures from raw sequencing data was described previously [19,20]. The miRNA sequencing data have been deposited in the GEO database under accession number GSE335697.

### 4.3. Functional Analysis of Ectopic Expression of miR-374b-5p in MCF-7 Cells

Transient transfection of miRNAs into cancer cells has been described previously [19,20]. The reagents used in this experiment are listed in Supplemental Table S1. The details regarding the functional analyses for cell proliferation and apoptosis in cancer cells were described in our previous paper [20,33].

### 4.4. Identification of Cancer-Promoting Genes Regulated by miR-374b-5p in MCF-7 Cells

*miR-374b-5p*-transfected MCF-7 cells were subjected to RNA sequencing to identify genes downregulated by *miR-374b-5p*. The RNA sequencing data have been deposited in the GEO database under accession number GSE337298. We searched for genes with *miR-374b-5p*-binding sequences within their 3′ UTRs using the TargetScanHuman ver. 8.0 database (https://www.targetscan.org/vert_80/, accessed on 15 September 2025). Then, the expression status of candidate genes in BrCa tissues was determined using the GEPIA2 database (http://gepia2.cancer-pku.cn, accessed on 20 November 2025). We used the OncoLnc database (http://www.oncolnc.org/, accessed on 20 November 2025) to investigate the relationships between the expression of the candidate genes and the prognosis of BrCa patients.

### 4.5. Regulation of FOXM1 Expression by miR-374b-5p

RNA and protein were extracted from *miR-374b-5p*-transfected MCF-7 cells, and *FOXM1* mRNA and protein levels were evaluated by PCR and Western blotting, respectively. The reagents used for PCR and Western blotting are listed in Supplemental Table S1. To determine whether *miR-374b-5p* binds directly to the 3′ UTR of *FOXM1*, we performed a dual-luciferase reporter assay. The *miR-374b-5p*-binding sequence within the *FOXM1* 3′ UTR was cloned into the psiCHEK2 vector (C8021; Promega, Madison, WI, USA). The cloned sequence is shown in Supplemental Figure S3. The procedure is described in our previous papers [19,20,33]. The reagents used for these analyses are listed in Supplemental Table S1.

### 4.6. The Combined Effects of Fulvestrant and a FOXM1 Inhibitor (FDI-6) in MCF-7 Cells

The effects of fulvestrant, as a selective ER downregulator/degrader, and FDI-6(S9689; Selleck Chemicals, Houston, TX, USA), as a *FOXM1* inhibitor, were assessed in MCF-7 cells. After treating MCF-7 cells with each inhibitor individually or with both inhibitors together, cell proliferation was analyzed using the XTT assay. The reagents used for these studies are listed in Supplemental Table S1.

### 4.7. Statistical Analysis

Statistical analyses were performed using GraphPad Prism software version 11.0.2 (GraphPad Software, San Diego, CA, USA). Differences between groups were assessed by Student’s *t*-test. Statistical significance was defined as *p* < 0.05.

## 5. Conclusions

A novel miRNA expression signature based on RNA sequencing was generated from MCF-7 cells treated with fulvestrant. Of the miRNAs affected, *miR-374b-5p* exhibited upregulation by fulvestrant treatment and acted as an antitumor miRNA in BrCa cells. In a search for *miR-374b-5p* target genes that are upregulated in BrCa and associated with patient prognosis, we identified *FOXM1*. Combining fulvestrant with a *FOXM1* inhibitor (FDI-6) significantly suppressed the proliferation of MCF-7 cells. This miRNA-based strategy may facilitate the identification of target molecules that enhance the antitumor efficacy of fulvestrant and contribute to the development of novel therapeutic regimens for ER-targeted endocrine therapy.

## Figures and Tables

**Table 1:** Upregulated miRNAs identified by RNA sequencing of Fulvestrant-treated MCF7 cells.

MicroRNA	miRBase Accession No.	Guide or Passenger Strand	Fold Change	Chromosome Location
*miR-664b-5p*	MIMAT0022271	Guide Strand	14.25	Xq28
*miR-1299*	MIMAT0022942	Guide Strand	13.59	5q35.3
*miR-132-3p*	MIMAT0000426	Guide Strand	8.25	17p13.3
*miR-139-5p*	MIMAT0000250	Guide Strand	6.87	11q13.4
*miR-550a-3-5p*	MIMAT0020925	Passenger Strand	6.56	7p14.3
*miR-4728-5p*	MIMAT0019849	Passenger Strand	5.93	17q12
*miR-579-5p*	MIMAT0026616	Passenger Strand	5.15	5p13.3
*miR-129-5p*	MIMAT0000242	Guide Strand	4.68	7q32.1
*miR-194-5p*	MIMAT0000460	Guide Strand	4.16	1q41
*miR-4748*	MIMAT0019884	Guide Strand	3.75	19p13.2
*miR-30a-3p*	MIMAT0000088	Passenger Strand	3.60	6q13
*miR-30a-5p*	MIMAT0000087	Guide Strand	3.45	6q13
*miR-139-3p*	MIMAT0004552	Passenger Strand	3.44	11q13.4
*miR-146b-5p*	MIMAT0002809	Guide Strand	3.12	10q24.32
*miR-6754-5p*	MIMAT0027408	Passenger Strand	3.04	11q13.4
*miR-6516-5p*	MIMAT0030417	Passenger Strand	2.93	17q25.2
*miR-3605-3p*	MIMAT0017982	Passenger Strand	2.91	1p35.1
*miR-184*	MIMAT0000454	Guide Strand	2.81	15q25.1
*miR-26a-5p*	MIMAT0000082	Guide Strand	2.75	3p22.2
*miR-5010-5p*	MIMAT0021043	Guide Strand	2.60	17q21.2
*miR-501-3p*	MIMAT0004774	Guide Strand	2.58	Xp11.23
*miR-30c-2-3p*	MIMAT0004550	Passenger Strand	2.44	6q13
*miR-181a-5p*	MIMAT0000256	Guide Strand	2.33	1q32.1
*miR-192-5p*	MIMAT0000222	Guide Strand	2.28	11q13.1
*miR-1260a*	MIMAT0005911	Guide Strand	2.28	14q24.3
*miR-1843*	MIMAT0039764	Guide Strand	2.25	1q25.1
*miR-765*	MIMAT0003945	Guide Strand	2.25	1q23.1
*miR-30c-5p*	MIMAT0000244	Guide Strand	2.19	1p34.2
*miR-374b-5p*	MIMAT0004955	Guide Strand	2.19	Xq13.2
*miR-3065-3p*	MIMAT0015378	Guide Strand	2.19	17q25.3
*miR-664a-5p*	MIMAT0005948	Guide Strand	2.15	1q41
*miR-374a-5p*	MIMAT0000727	Guide Strand	2.05	Xq13.2
*miR-182-5p*	MIMAT0000211	Guide Strand	2.03	7q32.2
*miR-29a-3p*	MIMAT0000086	Guide Strand	2.01	7q32.3
*miR-148b-3p*	MIMAT0000759	Guide Strand	2.01	12q13.13

**Table 2:** Candidate target genes regulated by *miR-374b-5p* in MCF7 cells.

Entrez Gene ID	Gene Symbol	Gene Name	Tissues Microarray Log_2_ Fold Change
440145	*MZT1*	mitotic spindle organizing protein 1	−1.48
4751	*NEK2*	NIMA-related kinase 2	−1.44
83540	*NUF2*	NUF2 component of NDC80 kinetochore complex	−1.43
3161	*HMMR*	hyalurona-mediated motility receptor	−1.43
80119	*PIF1*	PIF1 5′-to-3′ DNA helicase	−1.39
9928	*KIF14*	kinesin family member 14	−1.37
150468	*CKAP2L*	cytoskeleton-associated protein 2-like	−1.36
55789	*DEPDC1B*	DEP domain containing 1B	−1.34
1063	*CENPF*	centromere protein F	−1.26
55728	*N4BP2*	NEDD4 binding protein 2	−1.25
4277	*MICB*	MHC class I polypeptide-related sequence B	−1.25
2202	*EFEMP1*	EGF-containing fibulin extracellular matrix protein 1	−1.21
2305	*FOXM1*	forkhead box M1	−1.20
57405	*SPC25*	SPC25 component of NDC80 kinetochore complex	−1.20
79071	*ELOVL6*	ELOVL fatty acid elongase 6	−1.18
55635	*DEPDC1*	DEP domain containing 1	−1.18
23657	*SLC7A11*	solute carrier family 7 member 11	−1.17
10112	*KIF20A*	kinesin family member 20A	−1.15
157570	*ESCO2*	establishment of sister chromatid cohesion N-acetyltransferase 2	−1.15
890	*CCNA2*	cyclin A2	−1.13
653820	*FAM72B*	family with sequence similarity 72 member B	−1.13
29028	*ATAD2*	ATPase family AAA domain-containing 2	−1.13
23397	*NCAPH*	non-SMC condensin I complex subunit H	−1.13
79019	*CENPM*	centromere protein M	−1.12
64105	*CENPK*	centromere protein K	−1.12
728833	*FAM72D*	family with sequence similarity 72 member D	−1.11
3070	*HELLS*	helicase, lymphoid-specific	−1.10
80010	*RMI1*	RecQ-mediated genome instability 1	−1.08
760	*CA2*	carbonic anhydrase 2	−1.07
3281	*HSBP1*	heat shock factor-binding protein 1	−1.07
81610	*FAM83D*	family with sequence similarity 83 member D	−1.07
11130	*ZWINT*	ZW10 interacting kinetochore protein	−1.05
9768	*KIAA0101*	PCNA clamp-associated factor	−1.05
3005	*H1F0*	H1.0 linker histone	−1.04
1121	*CHM*	CHM Rab escort protein	−1.03
4886	*NPY1R*	neuropeptide Y receptor Y1	−1.03
3992	*FADS1*	fatty acid desaturase 1	−1.03
127933	*UHMK1*	U2AF homology motif kinase 1	−1.01

## Data Availability

Publicly available datasets were analyzed in this study. These data can be found here: [GSE335697/miRNA expression profiling of fulvestrant-treated MCF7 breast cancer cells]. The mRNA expression profiling data of MCF-7 breast cancer cells transfected with miR-374b-5p and miR-374a-5p are available in the GEO database under accession number [GSE337298/ Gene expression profiling of MCF7 breast cancer cells transfected with miR-374b-5p and miR-374a-5p].

## Supplementary Materials

The following supporting information can be downloaded at https://www.mdpi.com/article/10.3390/ijms27156787/s1.
