# Supplementary material for: MicroRNA Signatures of Fulvestrant-Treated Luminal Breast Cancer Cells: Identification of Therapeutic Targets Regulated by miR-374b-5p
Source: Int J Mol Sci. 2026 Jul 29;27(15):6787. doi: 10.3390/ijms27156787 (PMC13466626; doi:10.3390/ijms27156787)
Supplement: Supplementary file 1 [file ijms-27-06787-s001.zip › Revised Supplementary figure_ijms-4437504.pdf]

S-Figure S1

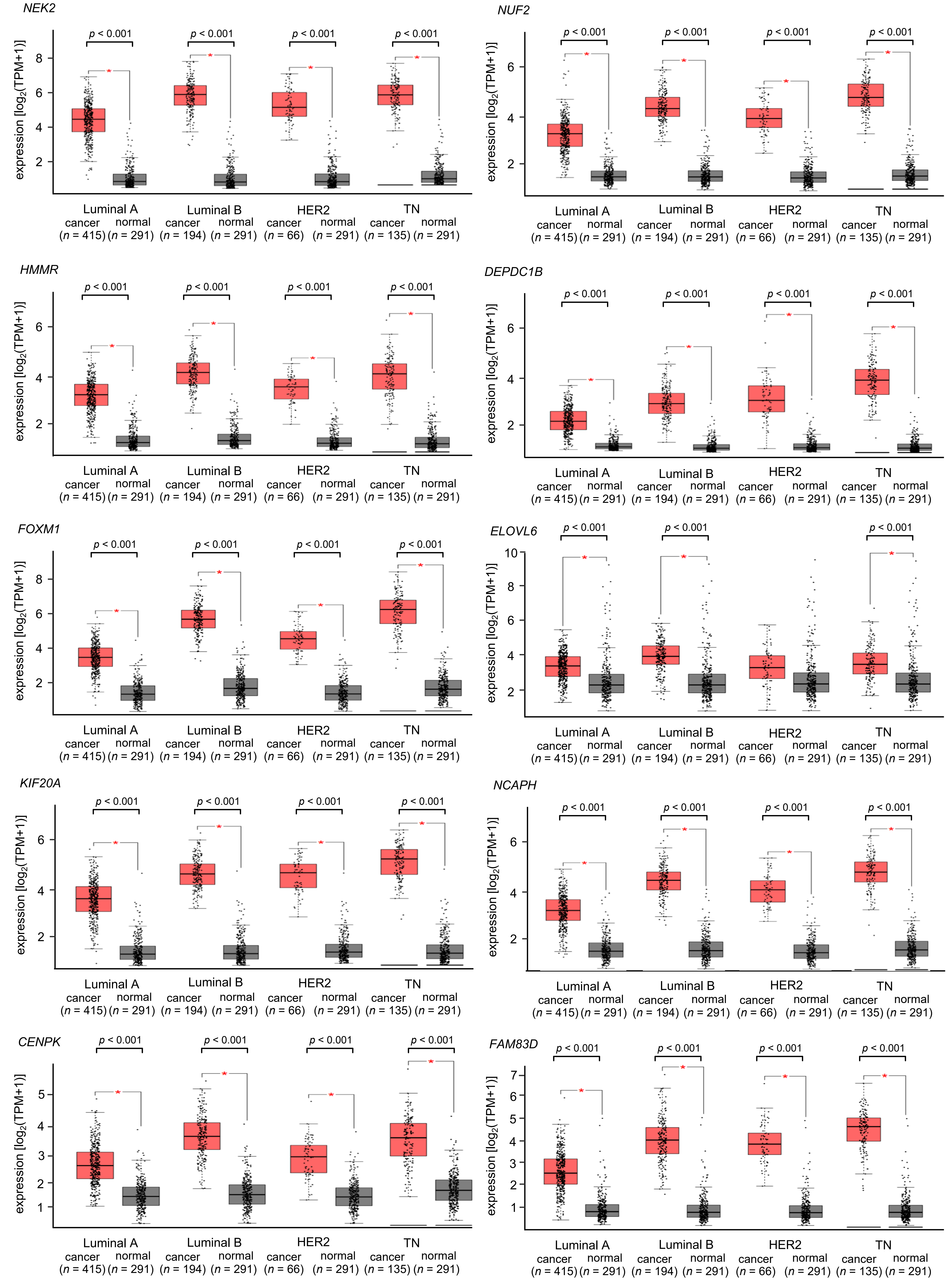

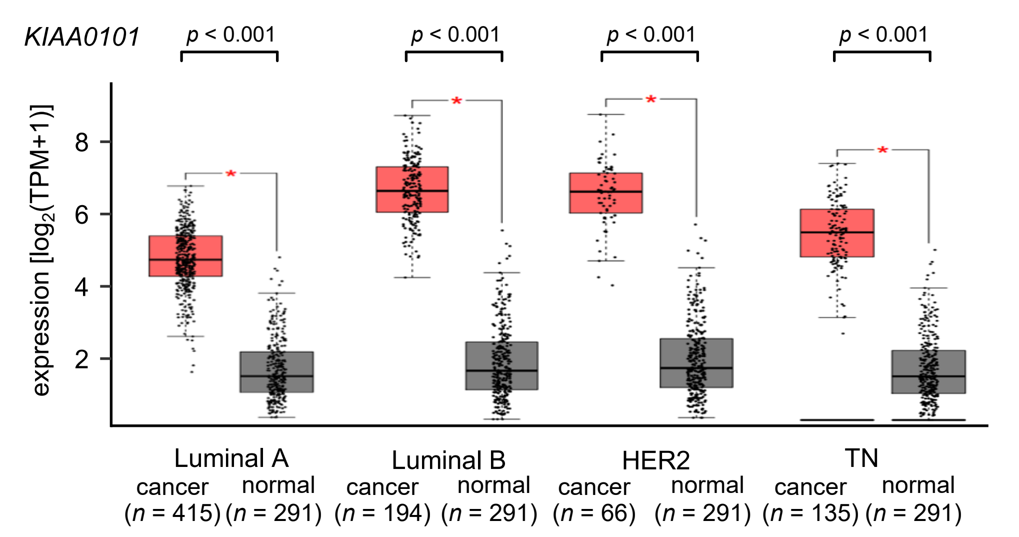

Supplemental Figure S1
Expression levels of the 11 genes (*NEK2, NUF2, HMMR, DEPDC1B, FOXM1, ELOVL6, KIF20A, NCAPH, CENPK, FAM83D,* and *KIAA0101*) in each breast cancer subtype (luminal A, luminal B, HER2-positive, and triple negative (TN)).

S-Figure S2

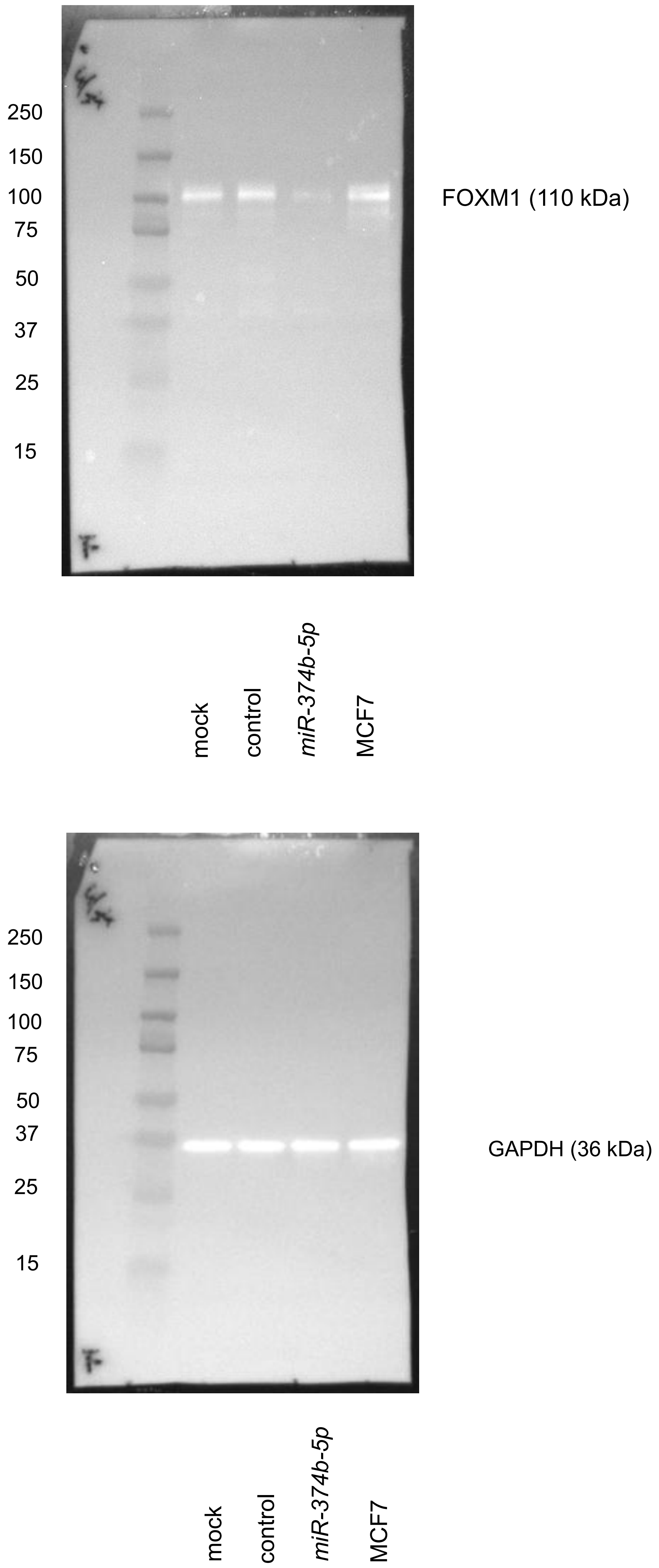

Supplemental Figure S2
Full-size Western blot of FOXM1 in MCF-7 cells transfected with *miR-374b-5p*.

Supplemental Figure S3

FOXM1 3'UTR

```
ctccaaggct cagtgcaccc caagcctctg agtgaggaca gcaggcaggg actgttctgc
tcctcatagc tccctgctgc ctgattatgc aaaagtagca gtcacaccct agccactgct
gggaccttgt gttccccaag agtatctgat tcctctgctg tccctgccag gagctgaagg
gtgggaacaa caaaggcaat ggtgaaaaga gattaggaac cccccagcct gtttccattc
tctgcccagc agtctcttac cttccctgat ctttgcaggg tggtccgtgt aaatagtata
aattctccaa attatcctct aattataaat gtaagcttat ttccttagat cattatccag
agactgccag aaggtgggta ggatgacctg gggtttcaat tgacttctgt tccttgcttt
tagttttgat agaagggaag acctgcagtg cacggtttct tccaggctga ggtacctgga
tcttgggttc ttcactgcag ggacccagac aagtggatct gcttgccaga gtcctttttg
cccctccctg ccacctcccc gtgtttccaa gtcagctttc ctgcaagaag aaatcctggt
taaaaaagtc ttttgtattg ggtcaggagt tgaatttggg gtgggaggat ggatgcaact
gaagcagagt gtgggtgccc agatgtgcgc tattagatgt ttctctgata atgtccccaa
```

Vector insert sequence

Wild-type

```
aattctccaa attatcctct aattataaat gtaagcttat ttccttagat cattatccag
```

Deletion-type

```
aattctccaa attatcctct a-------at gtaagcttat ttccttagat cattatccag
```

Supplemental Figure S3
The *miR-374b-5p*-binding sequence located in the 3' UTR of the *FOXM1* gene used for cloning.
